# Genetic Determinants for Pyomelanin Production and Its Protective Effect against Oxidative Stress in *Ralstonia solanacearum*

**DOI:** 10.1371/journal.pone.0160845

**Published:** 2016-08-11

**Authors:** Shabir Ahmad, Seung Yeup Lee, Hyun Gi Kong, Eun Jeong Jo, Hye Kyung Choi, Raees Khan, Seon-Woo Lee

**Affiliations:** Department of Applied Biosciences, Dong-A University, Busan, 49315, Republic of Korea; Soonchunhyang University, REPUBLIC OF KOREA

## Abstract

*Ralstonia solanacearum* is a soil-borne plant pathogen that infects more than 200 plant species. Its broad host range and long-term survival under different environmental stress conditions suggest that it uses a variety of mechanisms to protect itself against various types of biotic and abiotic stress. *R*. *solanacearum* produces a melanin-like brown pigment in the stationary phase when grown in minimal medium containing tyrosine. To gain deeper insight into the genetic determinants involved in melanin production, transposon-inserted mutants of *R*. *solanacearum* strain SL341 were screened for strains with defective melanin-producing capability. In addition to one mutant already known to be involved in pyomelanin production (viz., strain SL341D, with disruption of the hydroxphenylpyruvate dioxygenase gene), we identified three other mutants with disruption in the regulatory genes *rpoS*, *hrpG*, and *oxyR*, respectively. Wild-type SL341 produced pyomelanin in minimal medium containing tyrosine whereas the mutant strains did not. Likewise, homogentisate, a major precursor of pyomelanin, was detected in the culture filtrate of the wild-type strain but not in those of the mutant strains. A gene encoding hydroxyphenylpyruvate dioxygenase exhibited a significant high expression in wild type SL341 compared to other mutant strains, suggesting that pyomelanin production is regulated by three different regulatory proteins. However, analysis of the gene encoding homogentisate dioxygenase revealed no significant difference in its relative expression over time in the wild-type SL341 and mutant strains, except for SL341D, at 72 h incubation. The pigmented SL341 strain also exhibited a high tolerance to hydrogen peroxide stress compared with the non-pigmented SL341D strain. Our study suggests that pyomelanin production is controlled by several regulatory factors in *R*. *solanacearum* to confer protection under oxidative stress.

## Introduction

*Ralstonia solanacearum* is a soil-dwelling beta-proteobacterium that causes deadly wilt disease in over 200 plant species across 50 different plant families [1]. It causes disease in many commercially important plants, such as brown rot in potato, wilt in tomato, tobacco, and eggplant, and Moko disease in banana [2]. Based on its broad host range and wide geographical distribution, the pathogen holds the No. 2 position among the top ten plant pathogenic bacteria [3]. *R*. *solanacearum* invades host plants as a parasite and survives in soil or water as a saprophyte [4, 5]. Its broad host range and survival in soil or water for extended periods suggest that this pathogen may adopt a variety of mechanisms to confront both biotic and abiotic stress conditions.

Melanins are negatively charged hydrophobic macromolecules formed by the oxidative polymerization of phenolic or indolic compounds [6, 7]. These heterogeneous polymers are black, brown, or red in color, insoluble in organic and aqueous solutions, and resistant to concentrated acid [8, 9]. Based on their chemical properties, melanins can be divided into four classes: eumelanins, pheomelanins, allomelanins, and pyomelanins [10]. A significant number of bacteria, fungi, and different parasitic worms produce melanins [11]. Many microorganisms, such as *Pseudomonas* [12], *Aeromonas* [13, 14], *Azotobacter* [15], *Bacillus* [16, 17], *Streptomyces* [18], and *Vibrio* [19] species, produce different types of melanins.

Although melanins are not essential for the growth and survival of microorganisms, they provide some advantages to their producers to cope with different types of adverse challenges, such as UV radiation [20], toxic free radicals [21], oxidative stress [22], toxic heavy metals [23], iron reduction [24], and extreme cold and hot temperatures [25]. Melanin pigment also has a role in the expression of virulence factors in *Vibrio cholerae* [26]. Additionally, melanins are known to protect pathogenic microorganisms from the host immune response [27, 28].

**Fig 1:**
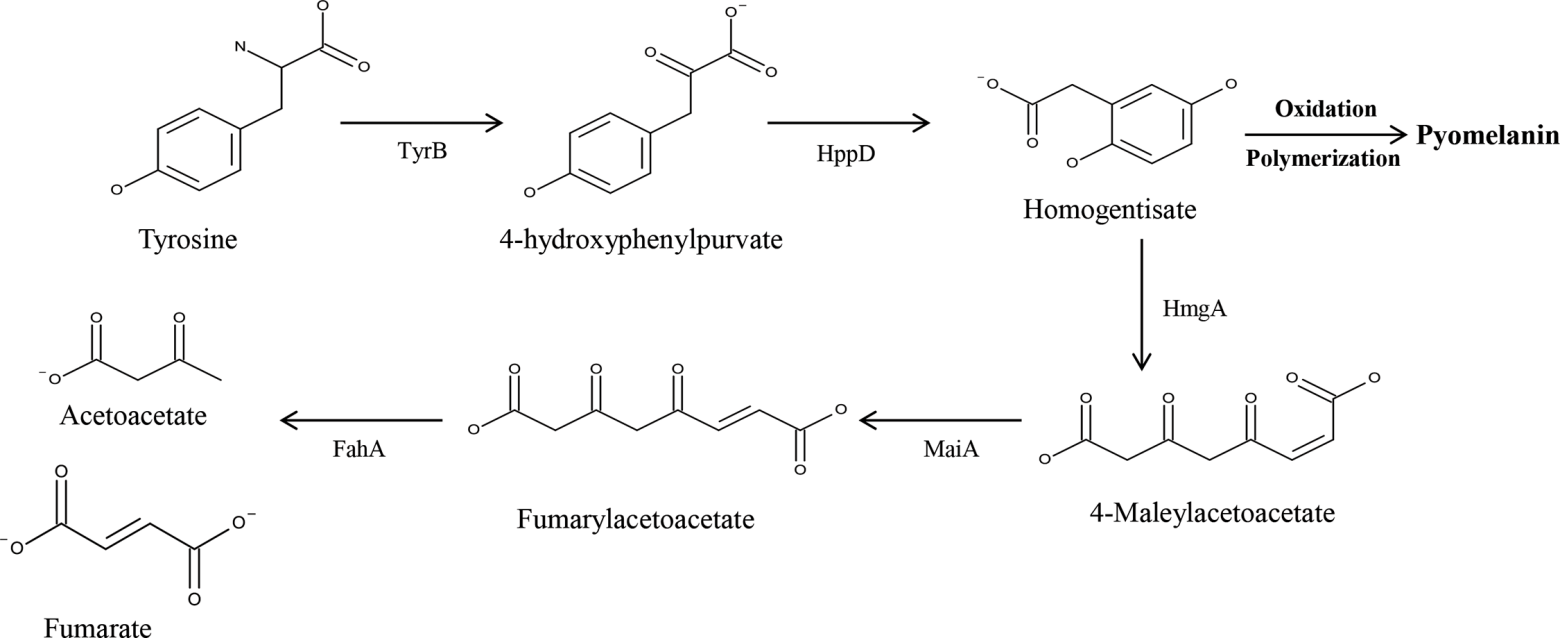
Metabolic pathway for tyrosine metabolism and pyomelanin production in *Ralstonia solanacearum* strain GMI1000. Tyrosine catabolic pathway linked with pyomelanin production. TyrB, aromatic amino acid aminotransferase; HppD, 4-hydroxyphenylpyruvate dioxygenase; HmgA, homogentisate dioxygenase; MaiA, maleylacetoacetate isomerase; FahA, fumarylacetoacetate hydrolase.

*R*. *solanacearum* produces a melanin-like brown pigment in the stationary phase in tyrosine-containing minimal medium. The genome of *R*. *solanacearum* carries genes for the melanin biosynthesis pathway [29] and also has two genes encoding tyrosinase [30]. The presence of a pyomelanin pathway (Fig 1) and multiple tyrosinases signifies the importance of melanin in this bacterium’s life cycle. Being a plant pathogen, *R*. *solanacearum* encounters oxidative challenge from its host during the plant infection process [31]. Therefore, we hypothesized that melanin confers a protective effect against the oxidative stress response of the host plant. The roles of the oxidative stress response regulator (OxyR) [32] and the DNA binding protein from starved cells (Dps) [33] in oxidative stress in *R*. *solanacearum* have previously been described, but there is scant knowledge about the physiological role of melanin in this species.

In this current study, we investigated the production of pyomelanin and its contribution to survival under oxidative stress in *R*. *solanacearum*. We also identified different genetic determinants that contribute to the regulation of pyomelanization in this bacterial species.

## Materials and Methods

### Bacterial strains and growth conditions

The bacterial strains and plasmids used in this study are listed in Table 1. The *Escherichia coli* strains were routinely cultured in Luria-Bertani (LB) medium at 37°C with shaking at 200 rpm. When necessary, antibiotics were added to the medium at the following concentrations: kanamycin, 50 μg/ml; tetracycline, 10 μg/ml; ampicillin, 100 μg/ml. *R*. *solanacearum* strain SL341 of race 1 (phylotype I) was used in these experiments [34] and was routinely cultured in casamino acid-peptone-glucose (CPG) medium [35] or CPG broth supplemented with 0.005% (w/v) 2,3,5-triphenyltetrazolium chloride (TTC) [36] and 1.5% agar. Selection of melanin-deficient mutants was performed on MG medium containing 1% mannitol, 0.2% l-glutamic acid, 0.05% KH_2_PO_4_, 0.02% NaCl, and 0.02% MgSO_4_ supplemented with 500 μg/ml tyrosine. The following antibiotic concentrations were used in the CPG, TTC, and MG media: kanamycin, 25 μg/ml; tetracycline, 10 μg/ml. Yeast extract-dextrose-CaCO_3_ (YDC) medium (containing 1% yeast extract, 2% glucose, 2% CaCO_3_, and 1.5% agar) was also used for triparental mating between the *R*. *solanacearum* mutants and *E*. *coli* donor and helper strains.

**Table 1:** Bacterial strains and plasmids used in study.

Bacterial strains and plasmids	Characteristics	Reference
***Ralstonia solanacearum***		
SL341	Wild-type, isolated from tomato plants, Race 1, Biovar4	34
SL341D	*hppD* (RSc3103):: Tn5; Kan^r^	This study
SL341DC	Trans conjugant of SL341Dcarrying pRKD, complementation of SL341D; Kan^r^, Tc^r^	This study
SL341S	*rpoS* (RSc1207):: Tn5; Kan^r^	This study
SL341SC	Trans conjugant of SL341Scarrying pRKS, complementation of SL341S; Kan^r^, Tc^r^	This study
SL341R	*oxyR* (RSc2690):: Tn5; Kan^r^	This study
SL341RC	Trans conjugant of SL341Rcarrying pRKR, complementation of SL341R; Kan^r^, Tc^r^	This study
SL341G	*hrpG* (RSp0852):: Tn5; Kan^r^	This study
SL341GC	Trans conjugant of SL341G carrying pRKG, complementation of SL341G; Kan^r^, Tc^r^	This study
***Escherichia coli***		
DH5α	F^-^ɸ80lacZDM15 D(*lacZYA*-*argF*)U169 deoR*recA*1 *endA*1 *hsdR*17 (rk^-^,mk^+^) phoA supE44λ- *thi*-1 *gyrA*96 *relA*1	39
HB101	F-*thi-1hsdS20* (rB^-^, mB^-^) *supE44 recA13 ara-14 leuB6 proA2 lacY1 galK2 rpsL20* (str^r^) *xyl-5 mtl-1*	41
**Plasmid**		
pUC119	Ap^r^; cloning vector	38
pGEM-T Easy	Ap^r^; T/A cloning vector	Promega
pGEMD	Ap^r^; pGEM-T Easy carrying 1.34 kb fragment of *hppD* gene of *R*. *solanacearum* SL341	This study
pGEMS	Ap^r^; pGEM-T Easy carrying 1.53 kb fragment of *rpoS* gene of *R*. *solanacearum* SL341	This study
pGEMR	Ap^r^; pGEM-T Easy carrying 1.24 kb fragment of *oxyR* gene of *R*. *solanacearum* SL341	This study
pGEMG	Ap^r^; pGEM-T Easy carrying 1.14 kb fragment of *hrpG* gene of *R*.*solanacearum* SL341	This study
pRK415	Tc^r^; PK2-derived broad host range cloning vector	40
pRKD	Tc^r^; pRK415 carrying 1.36 kb *EcoR1* fragment of pGEMD containing *hppD* gene of *R*. *solanacearum* SL341	This study
pRKS	Tc^r^; pRK415 carry 1.55 kb *EcoR1* fragment of pGEMS containing *rpoS* gene of *R*. *solanacearum* SL341	This study
pRKR	Tc^r^; pRK415 carrying 1.22 kb *EcoR1* fragment of pGEMR containing *oxyR* gene of *R*. *solanacearum* SL341	This study
pRKG	Tc^r^; pRK415 carrying 1.16 kb *Pst1* fragment of pGEMG containing *hrpG* gene of *R*. *solanacearum* SL341	This study
pRK2013	Km^r^; mobilization helper plasmid for triparental mating	42

Kan^r^, chromosomal kanamycin resistance; Ap^r^, ampicillin resistance; Tc^r^, tetracycline resistance; Km^r^, kanamycin resistance

### Screening of melanin mutants and identification of the transposon insertion site

A previously constructed transposon (Tn)-inserted mutant pool of SL341 [37] was screened to select mutants that produce no or less melanin relative to the wild-type SL341 strain. Mutants grown on MG agar plates were randomly selected and inoculated into 96-well culture plates containing 200 μl of minimal medium supplemented with tyrosine. The 96-well plates were incubated at 30°C in a shaking incubator at 200 rpm for 48 h. The mutant strains showing a reduced or non-pigmented phenotype relative to the wild type were selected and further confirmed on tyrosine-containing MG medium. The Tn insertion site in each mutant was identified according to a previously described method [37]. In brief, genomic DNA was extracted from each Tn mutant and randomly digested with *Sac1*. The digested DNA was ligated into pUC119 [38] that had been restricted with same restriction enzyme. After completion of ligation, the recombinant plasmid was transformed to *E*. *coli* DH5α [39] to select for transformants on LB agar plates containing kanamycin and ampicillin. Positive clones were selected from the plates and the recombinant plasmid was sequenced with Tn5-specific primers to identify the Tn-flanking DNA sequences.

For complementation of all the selected mutants, the wild-type copy of each mutated gene (the full length with its Shine-Dalgarno sequence) was amplified from genomic DNA of wild-type SL341 using each gene-specific primer (S1 Table). PCR amplification was performed according to the following program: an initial denaturation step at 95°C for 5 min; 30 cycles of denaturation at 95°C for 30 s, annealing at the specified temperature (S1 Table) for 30 s, and extension at 72°C for 1 min; and a final extension step at 72°C for 7 min. The amplified PCR product of each gene was first cloned into the pGEM-T Easy vector (Table 1), and the DNA sequence of each gene was confirmed by DNA sequencing. Each gene was restricted from the pGEM-T Easy vector with a specific restriction enzyme (Table 1) and subsequently subcloned into pRK415 under the *lac* promoter [40]. The recombinant plasmid carrying the corresponding gene (Table 1) was introduced into each mutant through triparental mating using *E*.*coli* HB101 [41] harboring pRK2013 as a helper plasmid [42]. Triparental mating was performed as previously described [37].

### Spectrophotometric analysis of melanin production

To investigate the melanin production by *R*. *solanacearum*, the SL341 and mutants strains were grown in minimal medium supplemented with tyrosine at 30°C with shaking at 200 rpm. A 1 ml culture sample was collected from the main culture of these strains after every 24 h and centrifuged at 13,000 ×*g* for 5 min. The cell-free supernatants were collected and their absorbance value was checked at 400 nm using a spectrophotometer (Beckman Coulter, Brea, CA, USA). The experiment was performed in triplicates.

### High-performance liquid chromatography analysis

High-performance liquid chromatography (HPLC) analysis of the culture filtrates of wild-type SL341 and its mutant strains was performed for homogentisate (HGA) detection. The bacterial strains were grown in minimal medium supplemented with tyrosine at 30°C in a shaking incubator at 200 rpm. Thereafter, 1 ml of culture sample was collected from the main culture of each strain at every 24 h. The samples were centrifuged at 13,000 ×*g* for 5 min and the supernatants were collected and filtered through a sterilized membrane filter (0.2 μm pore size; Corning, Tewksbury, MA, USA). Twenty microliters of the sample was injected onto an Agilent 1100 series HPLC system fitted with a Zorbax Eclipse Plus C18 column (5 μm particle size; 250 mm × 4.6 mm; Agilent, Santa Clara, CA, USA) and a photodiode array detector. The HPLC conditions for detection of tyrosine and HGA were as previously described [43]. In brief, for elution, water with 0.1% (v/v) trifluoroacetic acid was used as a solvent A, and acetonitrile with 0.1% (v/v) trifluoroacetic acid was used as a solvent B, at a flow rate of 1 ml/min. The following gradient was used: 8% solvent B for 12 min, gradient from 8% to 95% solvent B within 3 min, 95% solvent B for 1 min, gradient from 95% to 8% solvent B within 2 min, and finally 8% solvent B for 5 min. The total time for elution separation was 23 min. The tyrosine and HGA were detected at 280 nm and 290 nm, respectively. Commercially available tyrosine and HGA (Sigma-Aldrich, St. Louis, MO. USA) were used as the standards.

### Reverse transcription quantitative PCR study

The expression levels of *hppD* encoding 4- hydroxyphenylpyruvate dioxygenase, and *hmgA* encoding homogentisate dioxygenase were determined in the wild-type SL341 and mutant strains SL341D, SL341S, and SL341G, using the reverse transcription quantitative polymerase chain reaction (RT-qPCR). Total RNA was extracted from a 1 ml culture aliquot from each strain collected at different time points, using an RNA Hybrid-R extraction kit (GeneAll Bio Inc., Seoul, Korea). The RNA was eluted in 50 μl of RNase-free water and used directly as a template for cDNA synthesis using a Tetro cDNA synthesis kit (Bioline, London, UK), according to the manufacturer’s instructions. Before cDNA synthesis, each RNA sample was treated with DNaseI to remove any residual traces of DNA contamination. The cDNA concentration and purity of each sample were measured on a NanoDrop 2000 spectrophotometer (Thermo Scientific, Wilmington, DE, USA). The samples were normalized to the V3 region of the reference 16S rRNA gene of *R*. *solanacearum* SL341, and amplification reactions were performed using a CFX384 Real Time system (Bio-Rad, Hercules, CA, USA). The primers used for the RT-qPCR amplification reaction are given in (S1 Table.). Each reaction mixture contained SYBR Green Supermix (Bio-Rad), 4 μl of diluted cDNA template, 10 μM of both forward and reverse primers, and RNase-free water. The thermal cycling included two reaction steps; an initial preheat for 3 min at 95°C, followed by 39 cycles of 95°C for 5 s, 55°C for 10 s, and 72°C for 35 s. The RT-qPCR data were displayed using the CFX Manager ver. 3.1 software. Each reaction was performed in triplicates. The RT-qPCR results for the individual genes were evaluated using the iCycler iQ Real-Time PCR detection system (Bio-Rad). The C(t) values of the RT-qPCR products of each gene were used to determine the target cDNA concentration, based on relative comparison with the V3 gene expression. Tukey’s multiple tests were used to compare the expression levels of *hppD* and *hmgA* in wild-type SL341 and in the mutant strains at the different incubation times.

### *In vitro* oxidative stress assay

Oxidative stress survival of *R*. *solanacearum* was performed by adding hydrogen peroxide to the bacterial cultures. The wild-type SL341 and SL341D mutant strain were grown in minimal medium with and without tyrosine in a 30°C shaking incubator at 200 rpm. A 1 ml sample was collected from each culture after 72 h growth and centrifuged at 12,000 ×*g* for 5 min. The supernatants were discarded and the bacterial pellets were resuspended in 1 ml of sterile water. Viable cells in each sample were determined by culturing serial dilutions of the sample suspension on TTC agar plates. To evaluate the tolerance to the oxidative stress, hydrogen peroxide at a final concentration of 5 mM, 15 mM, 30 mM, and 50 mM were added to the remaining culture of each strain, in minimal medium with and without tyrosine, and the cultures were kept at 30°C in the shaking incubator for 1 h. Thereafter, the cultures were centrifuged, and the pellets were washed twice with sterile water and finally suspended in 1 ml of sterile water. Serially diluted cell suspensions of each culture were spread on TTC agar plates and kept at 30°C to determine the number of viable cells in each strain. The experiment was performed in triplicates. Tukey’s multiple range test was used to compare the number of viable cells in the treated strains of *R*. *solanacearum*.

## Results

### Selection of melanin-defective mutants of *R*. *solanacearum*

**Fig 2:**
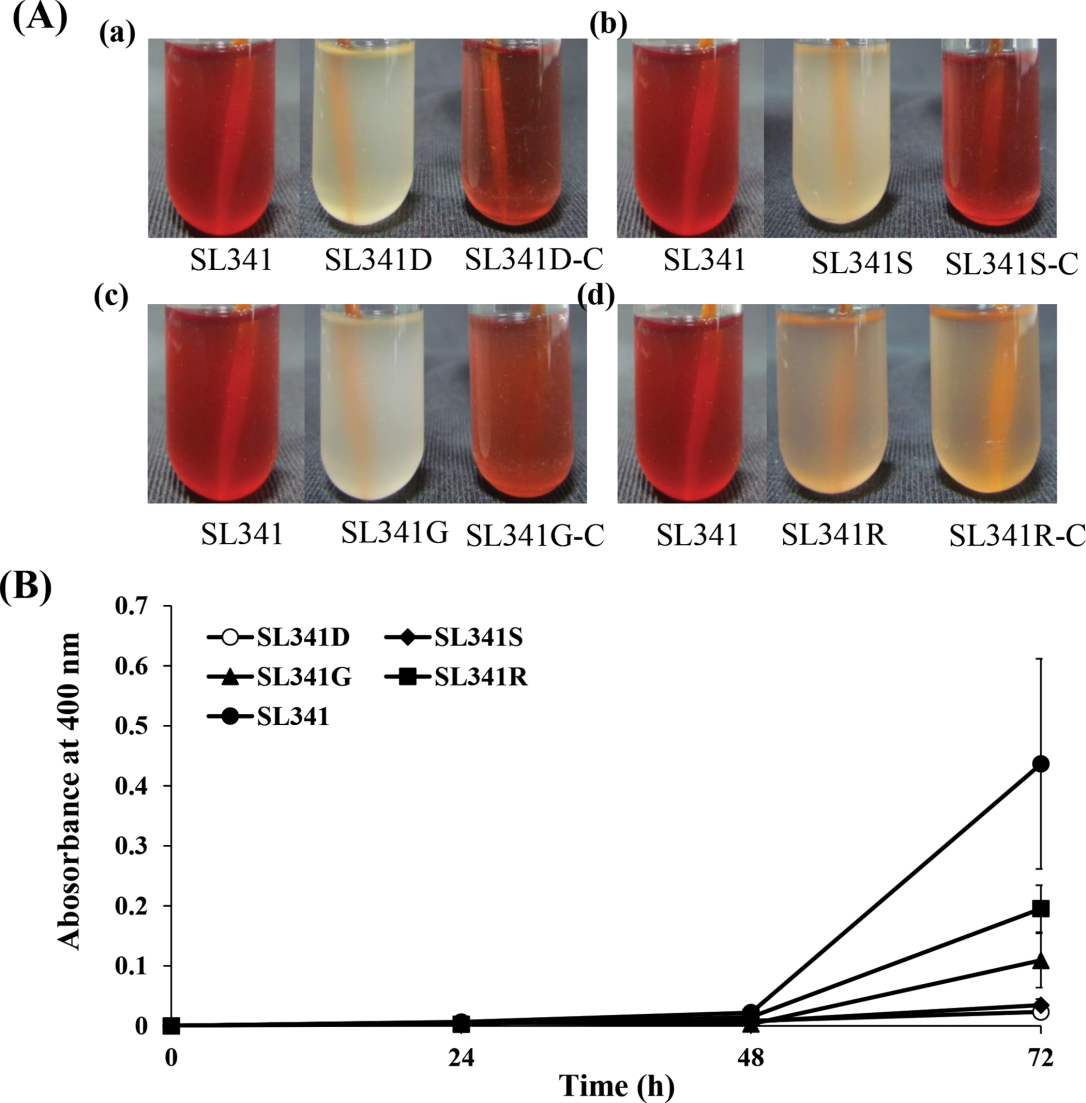
Complementation and melanin pigment production in *Ralstonia solanacearum* strains over time. **(A)** Complementation of pyomelanin defective mutants of *R*. *solanaccearum* SL341. Cultures of wild type SL341, pyomelanin deficient mutants, and complemented strains in minimal media containing tyrosine after 72 h growth. Each picture from left to right represent wild type, mutant and complemented strain, respectively. (a) Complementation of SL341D (Δ*hppD*) mutant strain restored its original phenotype. (b) Complementation of SL341S (Δ*rpoS*) mutant strain. (c) Complementation of SL341G (Δ*hrpG*) mutant strain. (d) Complementation of SL341R (Δ*oxyR*) mutant strain, which partially complemented the original phenotype. **(B)** Melanin pigment production in *Ralstonia solanacearum* strains over time. Pigment production was examined by measuring the absorbance of cell-free supernatant at 400 nm over time. Vertical bars represent the standard deviation of three biological replicates.

*R*. *solanacearum* SL341 produced blackish brown pigments similar to melanin in the medium supplemented with tyrosine (Fig 2A). The pigmentation was observed from most of the *R*. *solanacearum* strains tested; that is, in approximately 37 strains, including various phylotypes (data not shown). Using the race 1 (phylotype I) wild-type SL341 as a reference strain, we screened a total of 4,000 mutants from a Tn-based mutant pool of SL341 [37]. Based on visibly reduced pigmentation after 48 h of growth, 20 mutants without pigmentation were selected and further analyzed.

### Genetic determinants involved in pyomelanin production in *R*. *solanacearum*

To identify the disrupted gene in each mutant strain, selected subclones of each mutant were sequenced with Tn-specific primers. Based on the sequencing results and a BLAST search comparison, the 20 selected mutants showing no or less pigment production were classified into four groups because some of the mutants carried a mutation in the same gene. When we performed the complementation analysis with 20 mutants, only 11 of them were successfully complemented for pigmentation with their original gene. Therefore, we used 11 selected mutants for further analysis (S1 Fig). Among the selected mutants, one strain (SL341D) had a Tn insertion mutation in the 4-hydroxyphenylpyruvate dioxygenase (*hppD*) gene that had already been reported to be involved in the pyomelanin synthesis pathway (Fig 1, S1A Fig) [44], suggesting that *R*. *solanacearum* produces pyomelanin. Three other mutants (SL341S, SL341G, and SL341R) had Tn insertion mutations in regulatory genes (i.e., *rpoS*, *hrpG*, and *oxyR*, S1 Fig) that are novel candidates for involvement in pyomelanin production. In order to confirm whether or not the observed phenotype was associated with the disrupted gene, complementation studies were performed. Complete restoration of the original pigmentation was achieved in strains SL341D (*hppD*^-^), SL341S (*rpoS*^**-**^), and SL341G (*hrpG*^**-**^), whereas strain SL341R (*oxyR*^**-**^) was only partially complemented (Fig 2A). Therefore, the SL341D, SL341S, SL341G, and SL341R strains were chosen for further comparison studies with the wild-type strain.

### Quantitative analysis of melanin production

To investigate the effect of mutation on the level of pyomelanin produced in the four selected mutant strains of SL341, their culture supernatants were analyzed at different time intervals, along with that of the wild-type SL341 strain. The wild-type strain initially produced a small amount of pyomelanin at 24 h incubation, following which production increased slowly up to 48 h and then increased rapidly at 72 h incubation (Fig 2B). On the other hand, strain SL341D (carrying a Tn insertion in the *hppD* gene of the pyomelanin pathway) showed a very small amount of pigment production, even after 72 h growth. Similarly, SL341S revealed reduced pigment production even at 72 h incubation. On the other hand, SL341G and SL341R produced higher amounts of melanin at 72 h incubation although not comparable to the wild-type strain. This suggests that the regulation of pyomelanin production in *R*. *solanacearum* is likely by HrpG and OxyR.

### HPLC analysis for HGA detection

**Fig 3:**
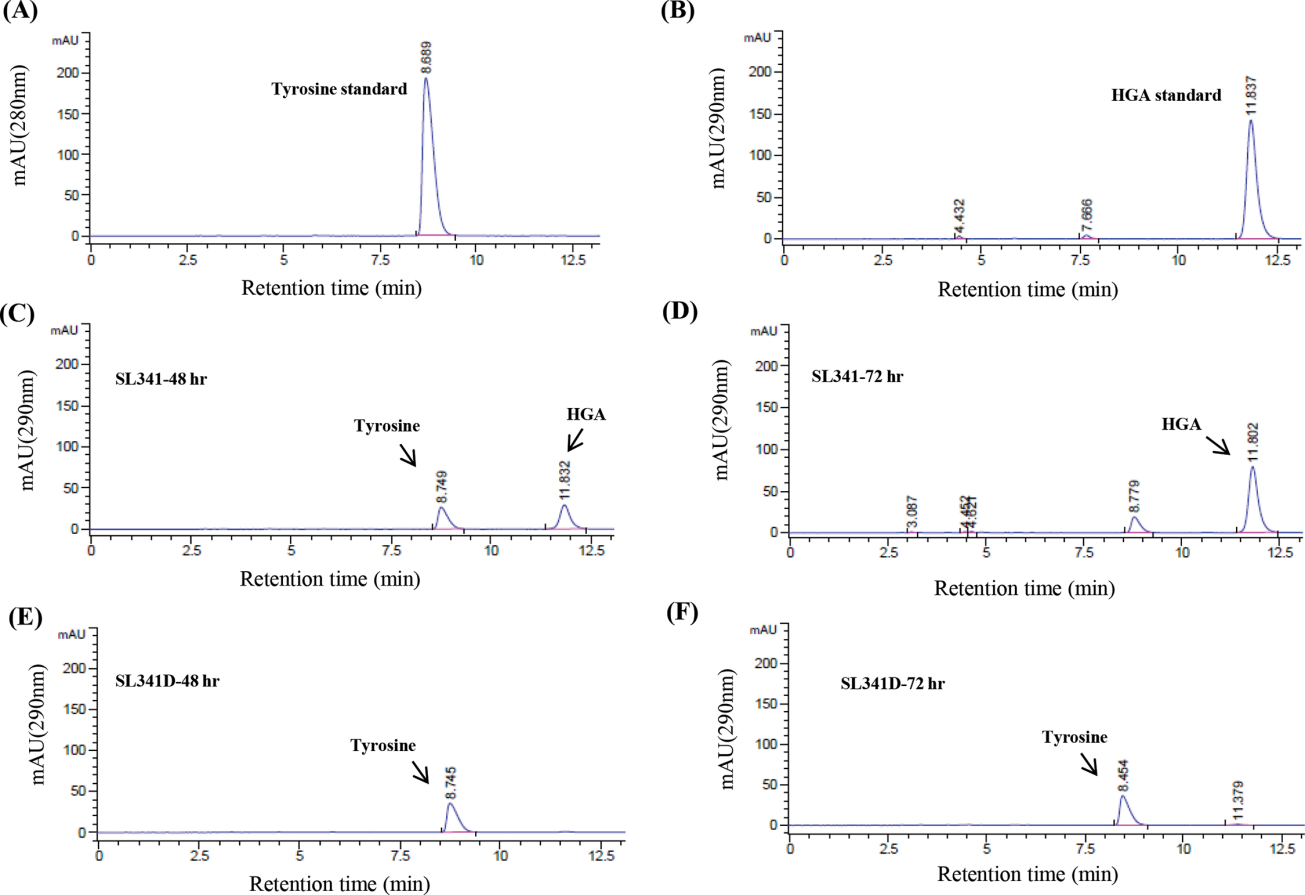
Detection of homogentisate from *Ralstonia solanacearum* strains by high-performance liquid chromatography. HPLC analysis of the culture filtrate of wild-type SL341 and SL341D over time for the detection of homogentisate (HGA), an intermediate in pyomelanin synthesis. (A, B) Tyrosine standard and HGA standard peaks. (C, D) Culture filtrate analysis of SL341 after 48 and 72 h growth, respectively. (E, F) Culture filtrate analysis of SL341D after 48 and 72 h growth, respectively.

To determine production of the pyomelanin intermediate HGA, HPLC analysis of the culture filtrates of the wild-type and mutant strains was performed at different time intervals. The standard tyrosine and HGA compounds generated single peaks at 10 min and 12.5 min, respectively, at their respective detection wavelength (Fig 3A and 3B). The wild type produced a significant amount of HGA after 48 h of growth (Fig 3C, S2 Fig), which remained stable and was still observed at 72 h culture (Fig 3D, S2 Fig) but not at 96 h (data not shown). In comparison, no HGA was observed in the cultures filtrates of SL341D (Fig 3E and 3F, S2 Fig) and the other three mutant strains over time (S2 and S3 Figs).

### Expression anlysis of *hppD* and *hmgA* in *R*. *solanacearum*

**Fig 4:**
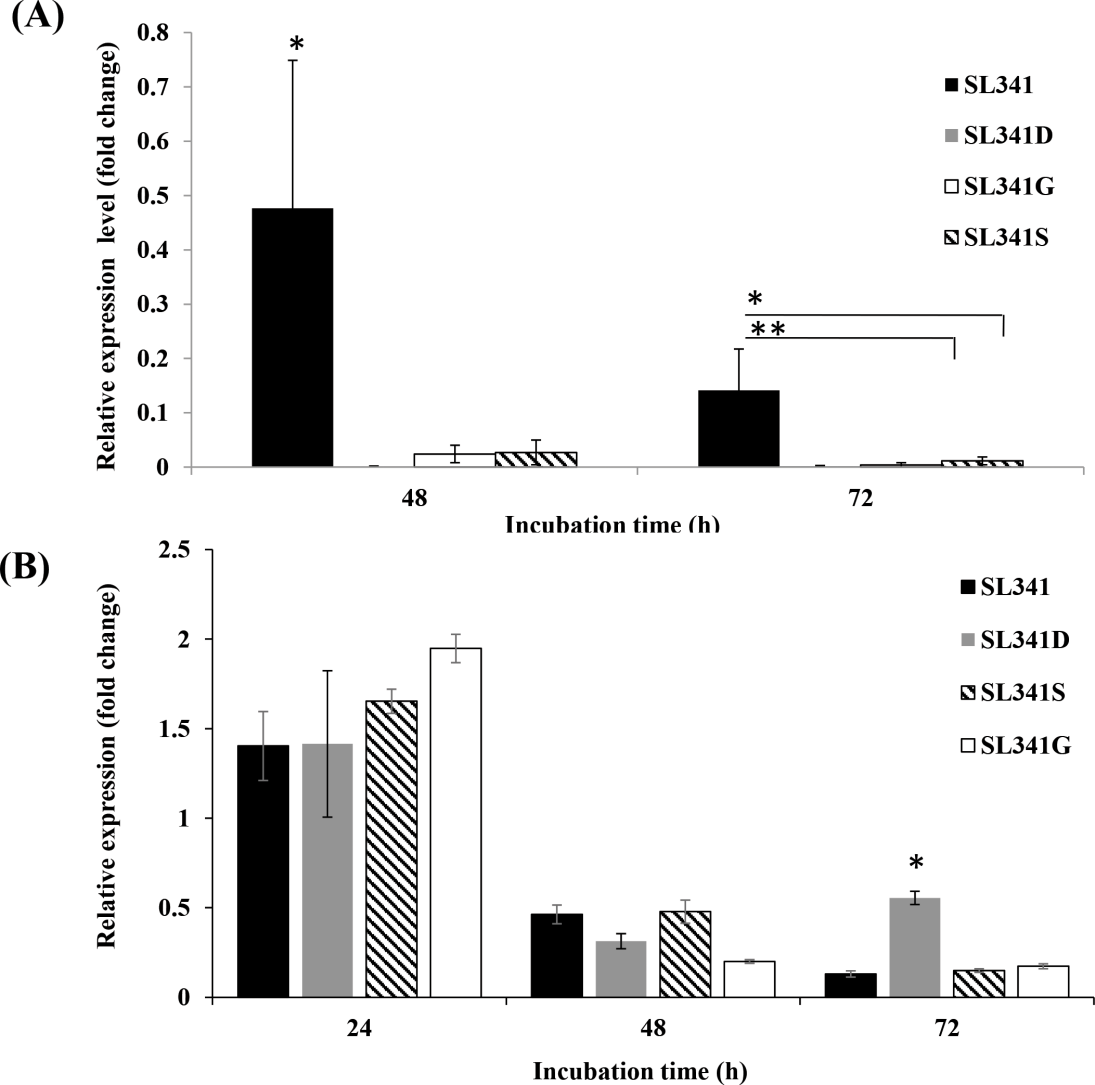
Relative expression of *hppD* gene and *hmgA* gene in *Ralstonia solanacearum* strains. Time-course mRNA expression of (A) *hppD* gene and (B) *hmgA* gene in strains SL341, SL341D, SL341S, and SL341G in minimal medium supplemented with tyrosine, as analyzed by RT-qPCR. The expression level of *hppD* and *hmgA* was normalized to that of the V3 region of the 16S rRNA gene as a reference. Vertical bars represent the standard deviation of three biological replicates. Asterisks above bar represent the significant difference among strains (“*”, *p*<0.05 and “**”, *p*<0.01 by Tukey’s test).

The expression of level of *hppD* and *hmgA* genes involved in pyomelanin biosynthesis pathway was investigated in SL341 and its mutants. The *hppD* gene encodes 4-hydroxyphenylpurvate dioxygenase which converts 4-hydroxyphenylpurvate into homogentisate, an intermediate in pyomelanin pathway, which is subsequently auto-oxidized and produces pyomelanin. The expression of *hppD* in wild type SL341 was significantly high (*p*<0.05) compared to its expression in selected mutant strains at 48 h incubation (Fig 4A). At 72 h incubation, the expression of *hppD* was also significantly higher in the wild type strain SL341 compared to the mutants strains SL341D and SL341G, and SL341S.

The *hmgA* gene encodes homogentisate dioxygenase to metabolize HGA, thus eliminating the pyomelanin intermediate. A time-course expression profile of this gene was investigated in SL341 and its mutants in minimal medium supplemented with tyrosine. The *hmgA* mRNA expression level in the wild type was relatively high at 24 h incubation, after which a consistent decrease in its expression was observed until 72 h (Fig 4B). The expression of *hmgA* in SL341D at 24 and 48 h was similar to that of the wild type, but the expression was significantly higher (*p*<0.05) at 72 h incubation compared with SL341 and the other mutant strains. In the case of both SL341S and SL341G, the gene expression level was high at 24 h incubation compared with the wild type and SL341D, but then decreased consistently until 72 h. Neither *hppD* nor *hrpG* were expressed at any time point in each corresponding mutant compared with the wild-type strain due to transposon insertion and gene inactivation (Data not shown). However, SL341S showed slight expression of *rpoS* at different incubation times (Data not shown).

### Pyomelanin role in hydrogen peroxide stress response

**Fig 5:**
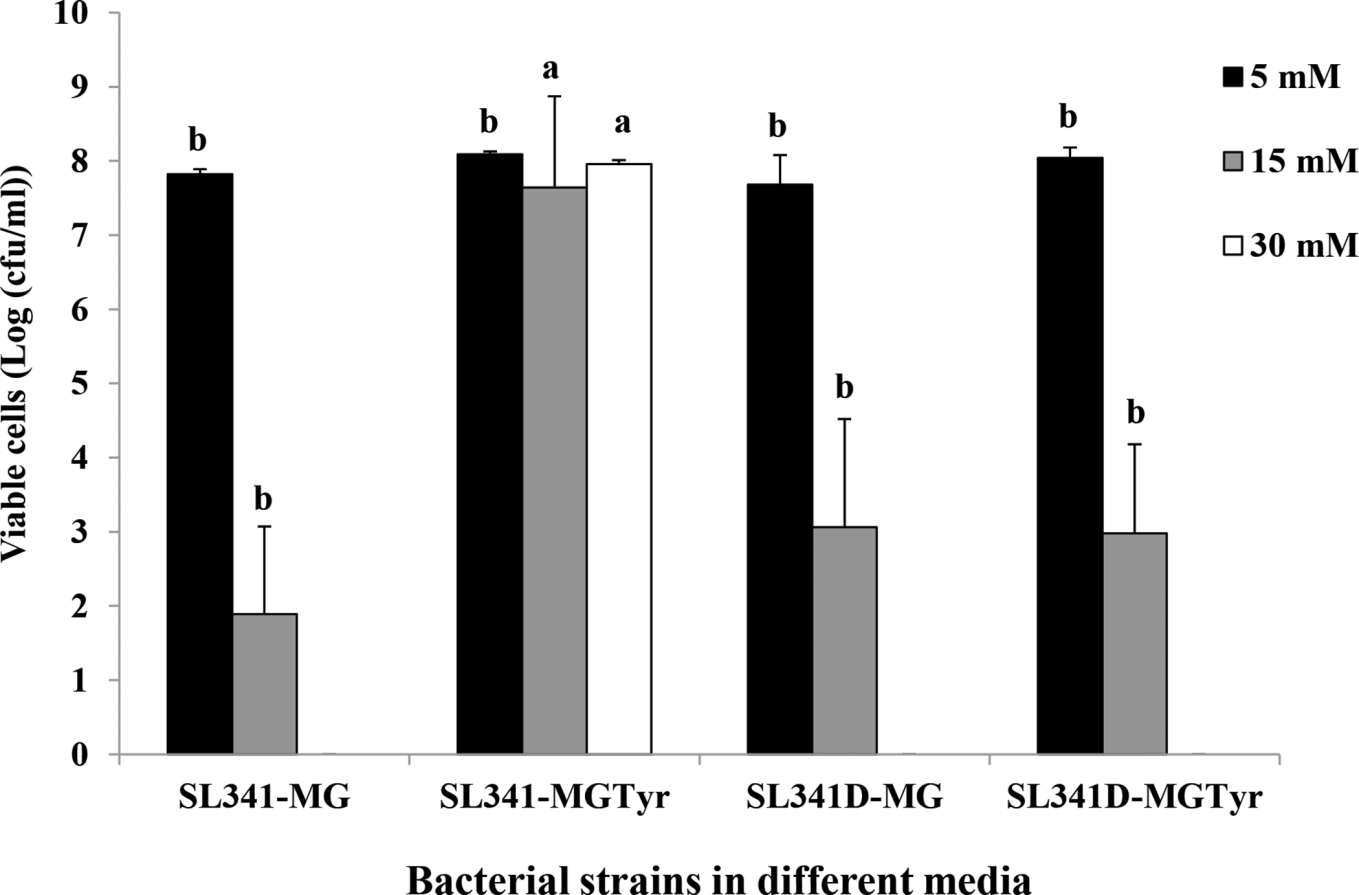
Survival of *Ralstonia solanacearum* strains SL341 and SL341D under hydrogen peroxide stress. Viable cell count of SL341 and SL341D grown in minimal medium with or without tyrosine and treated with different concentration of hydrogen peroxide. Each bar represents the mean from three biological replicates. Error bars indicate the standard deviation. Means in the same column followed by the same letter are not significantly different (*p*<0.05) by Tukey’s multiple range test, when compared among the same concentration of hydrogen peroxide treatment. Abbreviations: SL341-MG, SL341 strain grown in minimal growth medium; SL341-MGTyr, SL341 strain grown in minimal growth medium containing tyrosine; SL341D-MG; SL341D strain grown in minimal growth medium; SL341D-MGTyr, SL341D strain grown in minimal growth medium containing tyrosine.

Different concentrations of hydrogen peroxide were used to investigate the role of pyomelanin in the oxidative stress response in *R*. *solanacearum* wild-type SL341 and the *hppD*-disrupted mutant SL341D. The SL341 and SL341D grown in minimal media with and without tyrosine showed the similar bacterial survival pattern at 5 mM hydrogen peroxide concentration (Fig 5). The wild-type SL341, grown in minimal medium supplemented with tyrosine and thus producing pyomelanin, showed a significantly higher number of viable cells (*p*<0.05) than wild-type cells grown in minimal medium without tyrosine at 15 mM and 30 mM hydrogen peroxide. In the case of SL341D, there was no significant difference in the numbers of viable cells in the presence or absence of tyrosine, displaying relatively high cell numbers with 15 mM hydrogen peroxide but still significantly lower than wild-type cells grown in tyrosine-containing medium. At the level of 30 mM hydrogen peroxide treatment, only wild type strain SL341 exhibited hydrogen peroxide stress tolerance when those cells were grown in minimal media containing tyrosine (Fig 5). However, both wild type SL341 and the mutant SL341D grown with or without tyrosine did not exhibit any noticeable survival after hydrogen peroxide treatment at the concentration of 50 mM (data not shown).

## Discussion

Pyomelanin, a black-brown pigmented heterogeneous compound produced by a number of bacteria, fungi, and other organisms, has been associated with various physiological roles Although it has been extensively studied in human pathogens such as *Pseudomonas aeruginosa*, *Vibrio cholerae*, and *Aspergillus fumigatus* [22, 26, 45], there are no reports about the production and physiological role of this pigment in plant pathogenic bacteria. *R*. *solanacearum* produces a pyomelanin-like pigment, but the nature of this compound, its possible physiological role, and the genetic determinants involved in its production have not yet been elucidated. To the best of our knowledge, this is the first detailed study on pyomelanin production and regulation and its possible physiological role in *R*. *solanacearum*.

The use of Tn-based mutagenesis to identify the genetic determinants for melanin production was previously described in *Aeromonas media* WS and *P*. *aeruginosa* [13, 46]. The genes identified in these two studies were different from those of our current study, except for SL341D, which has a Tn insertion at gene *hppD* (reported to be involved in the pyomelanin biosynthesis pathway [44, 47]. These three studies indicate that the genetic determinants for melanin production and regulation are diverse among different organisms.

In our current study, we selected four mutants (including mutant SL341D) that had no or reduced pigmentation production ability. Specifically, the abolished pigment production in *hppD*-defective mutant SL341D provides genetic evidence of pyomelanin production by the wild-type *R*. *solanacearum*. Our current finding is contrary to a previous study, in which two genes encoding tyrosinases were identified in the genome of *R*. *solanacearum*, suggesting that this organism produces DOPA melanin [30]. We assumed that *R*. *solanacearum* produces pyomelanin, since the disruption of the *hppD* gene led to a complete loss of melanin production under our culture conditions. However, we concede that this bacterium may produce DOPA melanin under different medium conditions, although it is not clear why it would produce two different type of melanin in a tyrosine-supplemented medium.

Our result also revealed that *oxyR*, *rpoS*, and *hrpG*, which are involved in regulating the expression of genes under different stress responses and pathogenicity [48, 49, 50], may positively regulate pyomelanin production at the level of transcription in *R*. *solanacearum*. Melanin was previously reported to have different functions in different organisms [22, 26, 27, 28], and therefore, it may be possible that *R*. *solanacearum* will produce pyomelanin under different conditions for specific physiological roles. However, it will be premature at this stage to link the regulation of pyomelanin with a particular situation.

To support our assumption that the absence of pigment production by mutant SL341D indicates pyomelanin synthesis ability in wild-type *R*. *solanacearum*, we performed HPLC of the culture filtrates of SL341 and its mutant strains. Although it is quite difficult to detect HGA by HPLC because of its readily oxidizable nature, we were able to detected HGA in the culture filtrates of SL341 at 48 and 72 h, but not at 96 h, by which time it may have been completely oxidized (data not shown). As expected, the *hppD* mutation in the SL341D strain (Fig 1) impaired its pigment production ability, and hence HGA was not detected in its culture filtrate. In the case of the other mutants, although a small amount of pyomelanin was detected in their culture filtrates, we could not detect any HGA, possibly because it was in undetectable trace amounts only and/or metabolized further.

The relatively high expression of *hppD* in pyomelanin pathway in wild type SL341 compared to its mutant strains, suggests increased accumulation of HGA and subsequent high pyomelanin production. The expression of *hmgA* in SL341 decreased consistently over time, indicating that a small amount of HGA was converted into maleylacetoacetate in the tyrosine catabolic pathway, while most of the HGA was readily oxidized into pyomelanin. The expression of *hmgA* in SL341D and the other mutants was unexpected, as we assumed that HGA would not be produced in these non-pigment-producing mutants. A possible explanation is that *hmgA* expression is constitutive in *R*. *solanacearum* or that a deficiency of HGA may induce *hmgA* expression.

The melanin pigment has been associated with a variety of functions in different organisms [51]. Melanin from both natural and synthetic sources has efficient reactive oxygen species scavenging ability, protecting the producing organisms from their toxic effects [52]. Oxidative stress can affect the cell wall, nucleic acids, and lipids, and elicit various cellular responses in microorganisms [53]. Our current study showed that pyomelanin provides considerable tolerance to hydrogen peroxide stress. A similar protective effect of pyomelanin against hydrogen peroxide stress was reported in *Burkholderia cenocepacia* [54]. The pathogenic fungus *Cryptococcus neoformans* produces a melanin pigment that protects melanized cells from nitrogen- and oxygen-based antioxidants [55]. We speculated that pyomelanin would protect *R*. *solanacearum* from plant oxidative stress responses and provide additional protection from a plant’s initial immune response. However, we could not observe any difference in disease severity between the mutants and the wild-type strain by standard soil-soaking inoculation or petiole-injection inoculation (data not shown). Therefore, further studies are needed to elaborate the role of pyomelanin in *R*. *solanacearum*.

## Supporting Information

S1 Fig Transposon insertion site in different pyomelanin-deficient mutant strains of *R*. *solanacearum*. Transposon insertion site in the genome of the four pyomelanin-deficient mutants of *R*. *solanacearum*, (A) SL341D, (B) SL341S, (C) SL341G, (D) SL341R. The inverted triangle above each gene indicate Tn insertion site and the number above the triangle suggests the number of time mutant selected from different transposon pools.
(PPTX)

S2 Fig HPLC analysis for HGA production. HPLC analysis of culture filtrates of SL341, SL341G,SL341R, SL341S and SL341D for HGA production. Samples were collected from culture of each strain after every 24 h interval till 72 h. Error bar indicate standard deviation from 3 biological replicates.
(PPTX)

S3 Fig HPLC analysis for HGA detection. HPLC analysis of the culture filtrate of *R*. *solanacearum* mutants for the detection of HGA, an intermediate for pyomelanin synthesis. (A, B) Culture filtrate analysis of SL341S after 48 and 72 h growth, respectively, (C, D) SL341R culture filtrate analysis after 48 and 72 h growth, respectively (E, F) Culture filtrate analysis of SL341G after 48 and 72 h growth, respectively.
(PPTX)

S1 Table List of primers used in PCR reaction. (DOCX)
