# Supplementary material for: Genetic Determinants for Pyomelanin Production and Its Protective Effect against Oxidative Stress in Ralstonia solanacearum
Source: PLoS One. 2016 Aug 11;11(8):e0160845. doi: 10.1371/journal.pone.0160845 (PMC4981395; doi:10.1371/journal.pone.0160845)
Supplement: S1 Fig — Transposon insertion site in the genome of the four pyomelanin-deficient mutants of R. solanacearum, (A) SL341D, (B) SL341S, (C) SL341G, (D) SL341R. The inverted triangle above each gene indicate Tn insertion site and the number above the triangle suggests the number of time mutant selected from different transposon pools. (PPTX) [file pone.0160845.s001.pptx]

## Slide 1
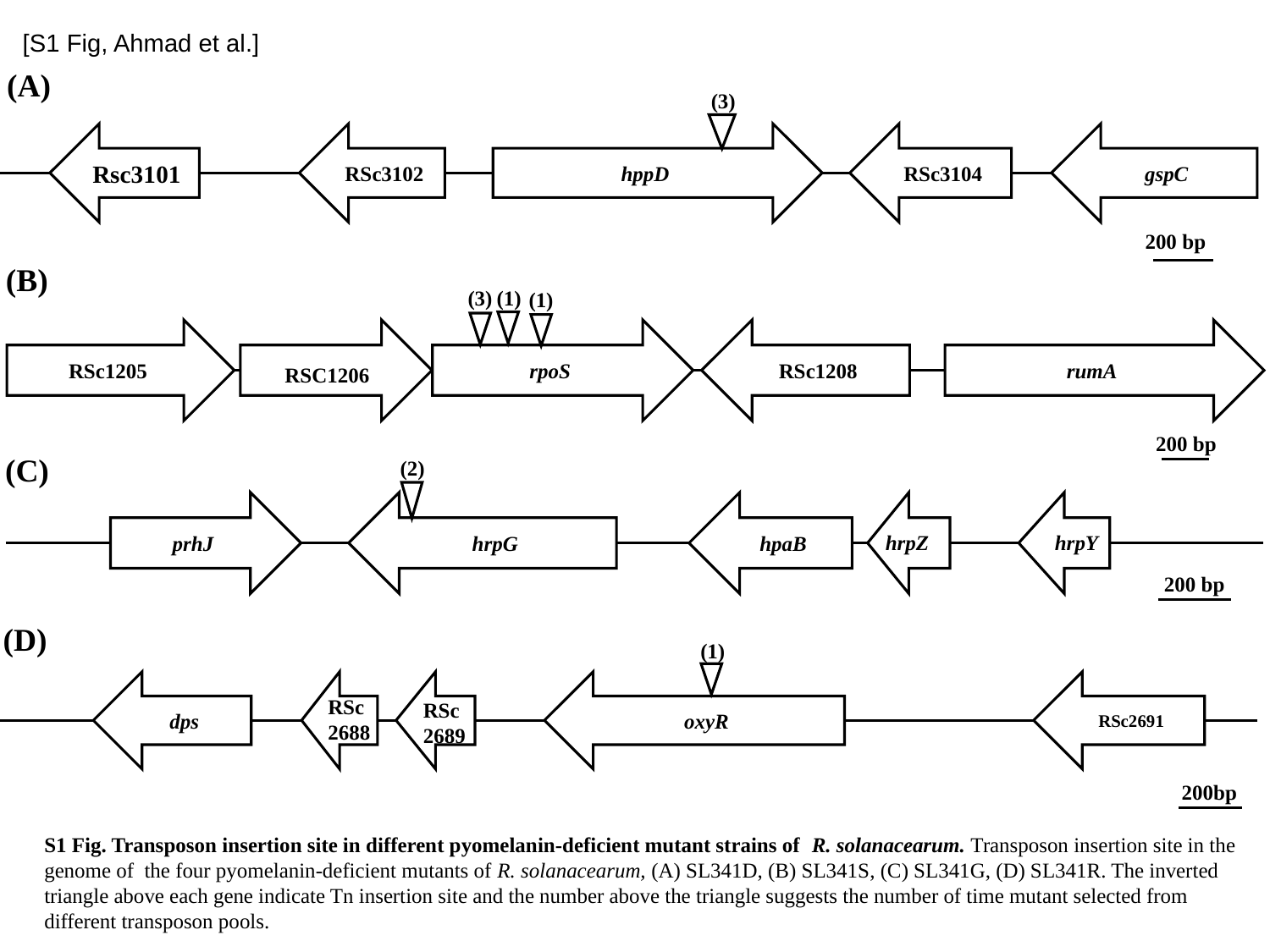

[S1 Fig, Ahmad et al.]
(A)
(3)
RSc3102
hppD
RSc3104
gspC
Rsc3101
200 bp
(B)
(3)
(1)
(1)
rpoS
RSc1205
 RSC1206
RSc1208
rumA
200 bp
(C)
(2)
prhJ
hrpG
hpaB
hrpZ
hrpY
200 bp
(D)
(1)
dps
oxyR
RSc2691
RSc
2688
RSc
2689
200bp
S1 Fig. Transposon insertion site in different pyomelanin-deficient mutant strains of R. solanacearum. Transposon insertion site in the genome of the four pyomelanin-deficient mutants of R. solanacearum, (A) SL341D, (B) SL341S, (C) SL341G, (D) SL341R. The inverted triangle above each gene indicate Tn insertion site and the number above the triangle suggests the number of time mutant selected from different transposon pools.
