# Supplementary material for: Genetic Determinants for Pyomelanin Production and Its Protective Effect against Oxidative Stress in Ralstonia solanacearum
Source: PLoS One. 2016 Aug 11;11(8):e0160845. doi: 10.1371/journal.pone.0160845 (PMC4981395; doi:10.1371/journal.pone.0160845)
Supplement: S2 Fig — HPLC analysis of culture filtrates of SL341, SL341G,SL341R, SL341S and SL341D for HGA production. Samples were collected from culture of each strain after every 24 h interval till 72 h. Error bar indicate standard deviation from 3 biological replicates. (PPTX) [file pone.0160845.s002.pptx]

## Slide 1
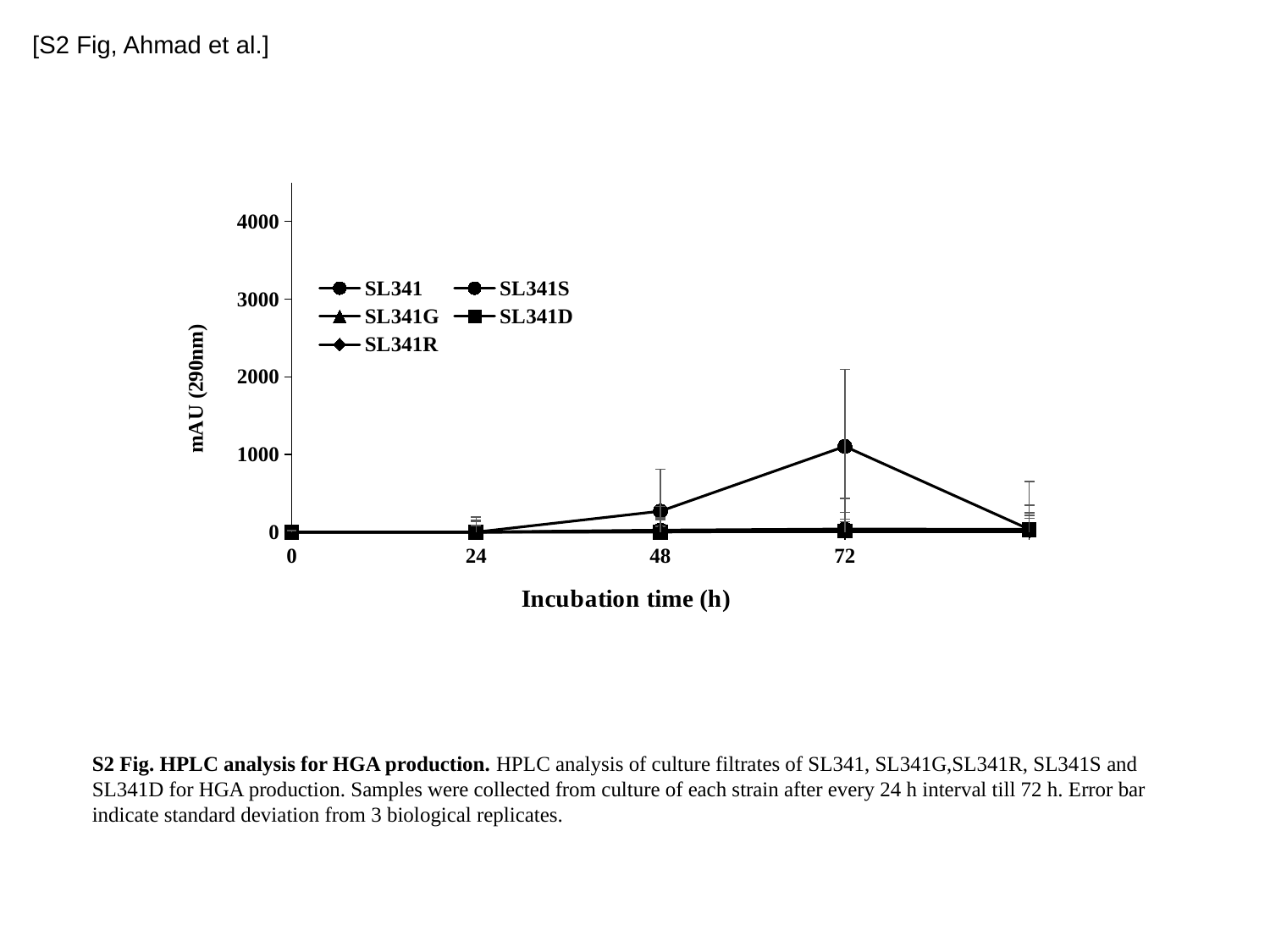

[S2 Fig, Ahmad et al.]
### Chart
| Category | SL341 | SL341S | SL341G | SL341D | SL341R |
|---|---|---|---|---|---|
| 0 | 0.0 | 0.0 | 0.0 | 0.0 | 0.0 |
| 24 | 0.0 | 0.0 | 0.0 | 0.0 | 0.0 |
| 48 | 268.514883333334 | 25.099059999999987 | 13.64075333333333 | 0.0 | 5.5551499999999985 |
| 72 | 1103.1962899999999 | 38.899496666666614 | 26.17773333333331 | 12.379720000000002 | 24.927030000000002 |
| | 35.75533000000003 | 35.329383333333325 | 27.762423333333288 | 32.675326666666635 | 16.04983 |S2 Fig. HPLC analysis for HGA production. HPLC analysis of culture filtrates of SL341, SL341G,SL341R, SL341S and SL341D for HGA production. Samples were collected from culture of each strain after every 24 h interval till 72 h. Error bar indicate standard deviation from 3 biological replicates.
