# Supplementary material for: Genetic Determinants for Pyomelanin Production and Its Protective Effect against Oxidative Stress in Ralstonia solanacearum
Source: PLoS One. 2016 Aug 11;11(8):e0160845. doi: 10.1371/journal.pone.0160845 (PMC4981395; doi:10.1371/journal.pone.0160845)
Supplement: S3 Fig — HPLC analysis of the culture filtrate of R. solanacearum mutants for the detection of HGA, an intermediate for pyomelanin synthesis. (A, B) Culture filtrate analysis of SL341S after 48 and 72 h growth, respectively, (C, D) SL341R culture filtrate analysis after 48 and 72 h growth, respectively (E, F) Culture filtrate analysis of SL341G after 48 and 72 h growth, respectively. (PPTX) [file pone.0160845.s003.pptx]

## Slide 1
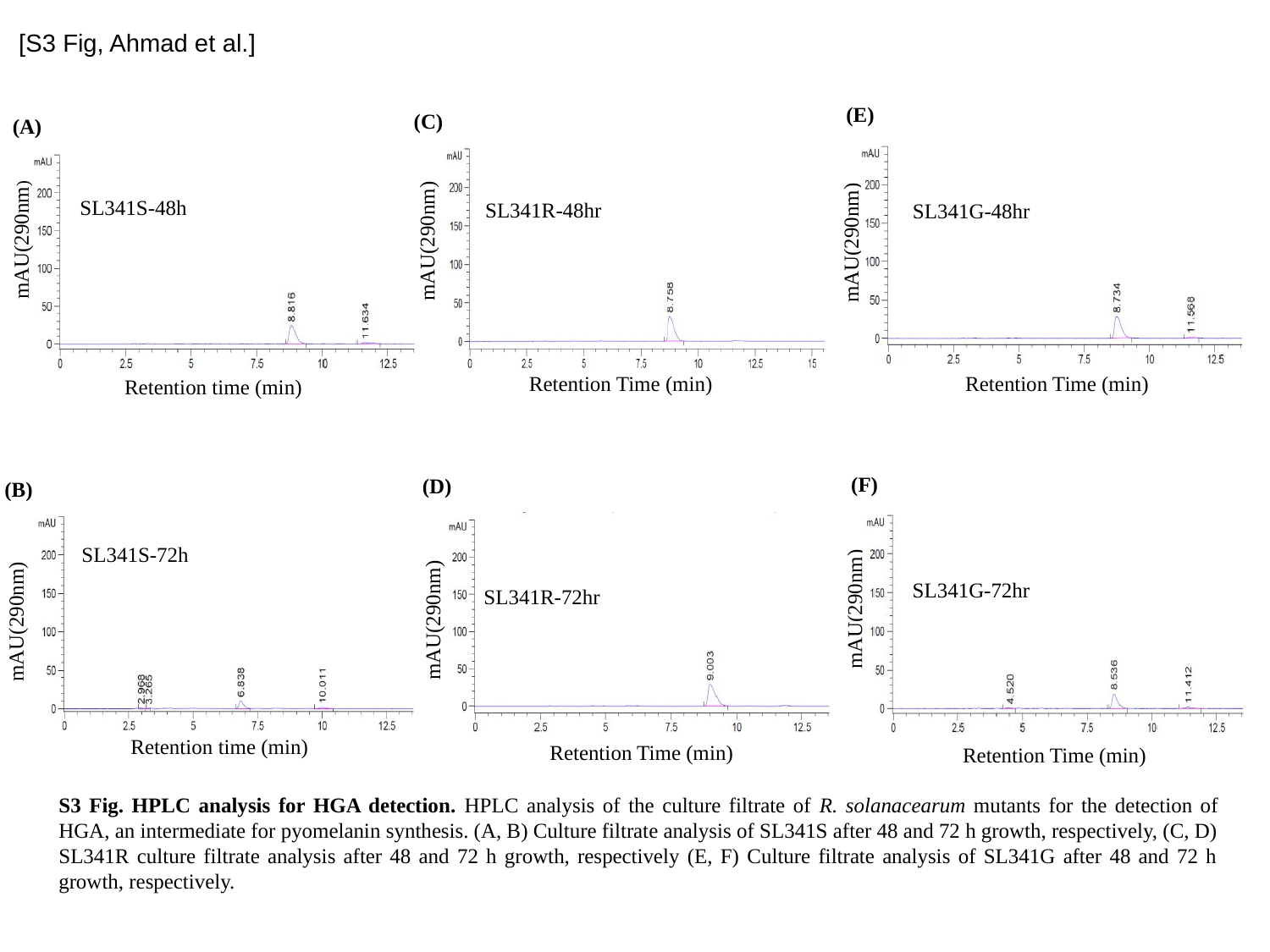

[S3 Fig, Ahmad et al.]
(E)
(C)
(A)
SL341S-48h
mAU(290nm)
Retention Time (min)
mAU(290nm)
SL341G-72hr
Retention Time (min)
SL341G-48hr
mAU(290nm)
Retention Time (min)
SL341R-48hr
Retention Time (min)
mAU(290nm)
Retention time (min)
SL341S-72hr
mAU(290nm)
Retention time (min)
(F)
(D)
(B)
SL341R-72hr
mAU(290nm)
Retention Time (min)
(D)
SL341S-72h
S3 Fig. HPLC analysis for HGA detection. HPLC analysis of the culture filtrate of R. solanacearum mutants for the detection of HGA, an intermediate for pyomelanin synthesis. (A, B) Culture filtrate analysis of SL341S after 48 and 72 h growth, respectively, (C, D) SL341R culture filtrate analysis after 48 and 72 h growth, respectively (E, F) Culture filtrate analysis of SL341G after 48 and 72 h growth, respectively.
