# Supplementary material for: Genetic Determinants for Pyomelanin Production and Its Protective Effect against Oxidative Stress in Ralstonia solanacearum
Source: PLoS One. 2016 Aug 11;11(8):e0160845. doi: 10.1371/journal.pone.0160845 (PMC4981395; doi:10.1371/journal.pone.0160845)
Supplement: S1 Table — (DOCX) [file pone.0160845.s004.docx]

**(Supplementary Information)**

**S1 Table. List of primers used in PCR reaction**

| **Gene** | **Primer (5’-3’)** | **Annealing**  **temperature** | **Product size** |
| --- | --- | --- | --- |
| **PCR Primer for Complementation** |  |  |  |
| *hppD* | F: TCTTGCGGTCGATCTTGTCC  R: ACACTCCCATGAAAAACGCG | 60℃ | 1,345bp |
| *rpoS* | F: TGATCCACGTTGGACCCTTG  R: CCTGCGATTTCATGCTGGTC | 60℃ | 1,536 bp |
| *oxyR* | F: ATATGGATCCCGCGCCCGAATATATCGTCT  R: ATATGGATCCGGTCGATTGCTGTCATCGGT | 60℃ | 1,245bp |
| *hrpG* | F: ACTGGCGGGACAACATACTG  R: GCATCAGCTTAAATCGCGGG | 60℃ | 1,149 bp |
| **Primer for RT-qPCR** |  |  |  |
| *hmgA* | F: TCAGGCCCATGAACTCGC  R: GCGCCTGTTCAACACCATC | 60℃ | 200bp |
| *hppD* | F: ATCAACGAGGAAGGGACGGA  R: CCGGGGATCCGCTTGTC | 60℃ | 199 bp |
| V3 region of 16S rRNA | F: ACTCCTACGGRAGGCAGCAG  R: ATTACCGCGGCTGCTGG | 60℃ | 190 bp |
